# Peril in the Pipeline: Unraveling the threads of PFAS contamination in U.S. drinking water systems

**DOI:** 10.1371/journal.pone.0299789

**Published:** 2024-04-04

**Authors:** Nabin B. Khanal, Levan Elbakidze

**Affiliations:** Davis College of Agriculture, Natural Resources & Design, West Virginia University, Morgantown, WV, United States of America; Lahore University of Management Sciences, PAKISTAN

## Abstract

We examined the spatial distribution of Per- and Polyfluoroalkyl Substances (PFAS) in the US drinking water and explored the relationship between PFAS contamination, public water systems (PWS) characteristics, and socioeconomic attributes of the affected communities. Using data from the EPA’s third Unregulated Contaminant Rule, the Census Bureau, and the Bureau of Labor Statistics, we identified spatial contamination hot spots and found that PFAS contamination was correlated with PWSs size, non-surface raw water intake sources, population, and housing density. We also found that non-white communities had less PFAS in drinking water. Lastly, we observed that PFAS contamination varied depending on regional industrial composition. The results showed that drinking water PFAS contamination was an externality of not only some industrial activities but also household consumption.

## 1. Introduction

Per- and Polyfluoroalkyl Substances (PFAS) cause serious health problems, including cancer, hyperlipidemia, thyroid disease, immunodeficiency, ulcerative colitis, chronic kidney disease, coronary artery disease, hypertension, and reduced fertility [1–4]. Ninety-five percent of US adolescents and adults are exposed to PFAS, primarily through drinking water [5, 6]. Between 16 and 270 million people in the US rely on PFAS-contaminated drinking water [7, 8]. Therefore, it is important to understand the incidence and distribution of PFAS in public water systems (PWS). In this study, we identify spatial contamination clusters and explore the socioeconomic factors associated with elevated PFAS in the US drinking water.

PFAS are a group of more than nine thousand synthetic chemicals widely used in industrial processes and consumer goods for their stain, grease, water, and heat-resistant properties [9]. The use and production of PFAS dates to more than 70 years ago when they were first used for uranium separation in the Manhattan Project [10]. Since then, these substances have become ubiquitous due to their resistance to degradation, water solubility, and the ability to translocate easily from one system to another [5]. PFAS have been identified as toxic and added to the Toxic Release Inventory list under the National Defense Authorization Act [11]. In 2021, the Department of Energy (DOE) issued a Departmental policy that aimed to reduce or eliminate PFAS release from departmental operations [10]. Part of the DOE’s objectives is to identify and quantify Cold War era sources of PFAS, including uranium processing operations.

Detrimental health impacts of PFAS have not been well understood, documented and widely recognized until recently. It wasn’t until the EPA’s Health Advisories were revised in 2022 that the safe level thresholds of Perfluorooctanoic acid (PFOA), Perfluorooctane sulfonic acid (PFOS), and other PFAS in drinking water were significantly lowered, suggesting that even small exposure can have detrimental health impacts [12, 13]. The 2016 health advisories for PFOA and PFOS indicated that less than 70 ppt (Part Per Trillion) posed no health risks, while the 2022 advisory lowered the threshold to 0.004 and 0.02 ppt, respectively. The addition of GenX (Hexafluoropropylene Oxide Dimer Acid and its Ammonium Salt) and Perfluorobutane sulfonate (PFBS) to the list of hazardous PFAS further highlights the growing recognition of the dangers that these chemicals pose. As more research is done and the risks associated with PFAS become better understood, it is critical that an appropriate public policy is developed to reduce exposure and prevent further contamination of the environment and drinking water sources.

In response to growing public concerns, the EPA announced the PFAS strategic Roadmap in October 2021 [14]. The roadmap outlines the agency’s plans to protect the public and the environment from PFAS contaminants by minimizing discharge, identifying and removing these compounds from ecosystems, and designating PFOA and PFOS as hazardous compounds under Comprehensive Environmental Response, Compensation, and Liability Act (CERCLA). The EPA has also committed to conducting environmental and health toxicity assessments for other PFAS, including Perfluorobutanoic acid (PFBA), PFGxA, Perfluorohexanesulfonic acid (PFGxS), and Perfluorodecanoic Acid (PFDA) [14]. Additionally, the roadmap includes provisions to ensure that disadvantaged communities have access to PFAS mitigation solutions. To make progress towards these goals, one must understand the distribution of exposure to PFAS via drinking water, including regional contamination clusters, differences across public water system types, the vulnerability of certain communities, and socioeconomic factors associated with contamination.

Protecting drinking water from PFAS contamination is a complex challenge. First, PFAS substances are unregulated under the Safe Drinking Water Act (SWDA), which means that PWSs are not required to monitor and control PFAS in the water they supply. Second, although activated carbon filters can be used as a strategy to reduce PFAS in drinking water [15] and additional technological solutions are emerging, there is currently no cost-effective technology to decompose and/or remove all PFAS from the environment [15–18]. Third, the sources of contamination are not well understood, making it challenging to prevent future contaminations. Fourth, PFAS substances are persistent. They bioaccumulate and do not easily break down, which increases the risk of exposure and makes it difficult to remove these compounds from the environment.

Drinking water PFAS contamination can be framed as an economic externality. Externalities are present when market transactions affect third parties without compensation. Alternatively, economic externalities can be formulated as transaction outcomes where prices do not reflect full social opportunity costs [19–21]. The health and environmental impacts of PFAS are externalities from industrial activities and from household consumption. On the industrial side, PFAS can leak into the surrounding watersheds, which serve as raw water sources for local PWSs, from production operations that rely on these substances. If drinking water facilities lack appropriate treatment technologies, then elevated levels of PFAS in the intake source can result in contamination of public drinking water. Drinking water PFAS contamination can also be an externality from household consumption goods that contain these materials. For example, many household goods contain PFAS chemicals, which end up in local watersheds and sewage systems. If wastewater facilities lack appropriate technologies to remove PFAS before discharging treated waste, then raw water intake for PWS facilities can be contaminated. Subsequently, if PWS facilities are ill equipped to remove PFAS then finished drinking water is contaminated.

A detailed analysis of PFAS across US PWSs is imperative for developing effective drinking water contamination policies. Our objective in this study is to shed light on some of the key questions on the distribution and incidence of elevated PFAS in drinking water. How pervasive is PFAS in drinking water, and are there regional clusters of contamination? Does PFAS contamination depend on PWS characteristics like size and water sources? Are some communities more vulnerable than others? Is PFAS drinking water contamination driven by industrial production and/or final consumption? Is PFAS contamination fundamentally similar to SDWA (Safe Drinking Water Act) regulated pollutants requiring similar mitigation approaches?

Environmental Protection Agency (EPA) uses the Unregulated Contaminant Monitoring Rule (UCMR) to assess the presence of contaminants that do not have health-based standards under the SDWA. Every five years, the EPA UCMR program identifies 30 potentially harmful but unregulated contaminants and tests all large Public Water Systems (PWSs) that serve more than ten thousand people, as well as a subsample of smaller facilities. In the UCMR3 program, the EPA tested six per- and polyfluoroalkyl substances (PFAS): perfluorooctanesulfonic acid (PFOS), perfluorooctanoic acid (PFOA), perfluorononanoic acid (PFNA), perfluorohexanesulfonic acid (PFHxS), perfluoroheptanoic acid (PFHpA), and perfluorobutanesulfonic acid (PFBS) in 2014–2016. Water samples were collected at the entry points to the distribution systems by PWS operators and sent to EPA-approved labs to test for the presence of each PFAS [22, 23]. PFAS Minimum Reporting Levels (MRLs) ranged from 10 to 90 ng/L [23]. The MRLs were 10 ng/L for PFHpA, 20 ng/L for PFOA and PFNA, 30 ng/L for PFHxS, 40 ng/L for PFOS, and 90 ng/L for PFBS. PFAS below the MRLs were not reported.

PWSs deliver drinking water to 95% of the US population [24] and the UCMR3 program tested PWSs that serve 75% of the US population. UCMR3 tested 4,120 large PWSs that serve more than 10,000 consumers and randomly selected 800 small PWSs that serve 10,000 or fewer consumers in the US and its territories [23]. Cumulatively, this sample represents 79% of the U.S. PWS consumer base. Many PWSs have more than one water supply facility, and UCMR3 tested 15,195 facilities within the selected PWSs in the USA and its territories.

Several studies have utilized the UCMR3 PFAS data [7, 8, 25, 26]. Hu et al. [8] estimated that 16.5 million people were exposed to PFAS via drinking water. Andrews and Naidenko [7] and Cadwallader et al. [25] used UCMR3 and data from some states with lower MRLs than UCMR. Extrapolating their findings from the states in their samples, Andrews and Naidenko [7] estimated the exposure rate of between 18 and 80 million people if the MRL was 10 ng/L and higher, and over 200 million if the MRL was at or above 1 ng/L. However, neither study explored the relationship between contamination and community socioeconomic characteristics.

Guelfo & Adamson [26] used the UCMR3 data and investigated the co-occurrence of different types of PFAS and the relationship with PWS characteristics. They found that PFHpA, PFOA, and PFNA were dominant in surface water whereas PFOS, PFHxS, and PFBS were dominant in groundwater. Large PWSs were more vulnerable to contamination than small ones. However, they did not account for data censoring due to MRLs in UCMR3. They also did not include socioeconomic factors that may impact contamination. We expand on their study by including socioeconomic and industrial covariates and by controlling for data censoring.

Some of the potential sources of PFAS contamination in UCMR3 data are examined in Hu et al. [8]. Using 8-digit HUC (Hydrologic Unit Code) data, they examined the spatial correlation between drinking water PFAS content and major industrial sites, military fire training sites, aqueous film-forming foams certified municipal airports, and wastewater treatment plants. They found that military fire training sites were strong predictors of PFOS and PFHxS. They also reported that PFOA, PFOS, and PFHxS concentrations were correlated with wastewater treatment. None of the factors they examined predicted PFHpA concentration. To examine industrial sources of PFAS, they only included 16 sites that participated in the EPA’s 2010/2015 PFOA Stewardship program. We contribute by examining the association between drinking water PFAS and broader industrial categories using county scale GDP shares as indicators. We also extend Hu et al. [8] by identifying contamination hotspots, examining a wider set of socioeconomic factors, and accounting for the censoring of UCMR data.

We make significant contributions to the existing literature in three respects. First, we use a Tobit regression model that accounts for UCMR data censoring due to MRLs [27, 28]. Most of the previous studies do not explicitly account for UCMR data censoring in their estimations [8, 26]. Some studies supplement UCMR data with state data to address some of the censoring issues [7, 25]. However, state level data are inconsistent in terms of format, detail, water type (raw versus finished), and representativeness as some data come from targeted and others from non-targeted sampling efforts [25]. The Tobit model accounts for censoring in a systematic manner to produce unbiased and consistent parameter estimates with better predictive accuracy and valid statistical inference relative to the OLS estimation [27]. Second, we shed light on the relationship between PFAS contamination and PWS characteristics, community socioeconomic factors, and regional industrial composition. We use publicly available data from UCMR3, the Bureau of Labor Statistics (BLS), and the American Community Survey (ACS) 5-year estimate to identify socioeconomic and industrial characteristics that correlate with PFAS contamination. These data allow for a more expanded examination relative to prior literature. Third, we isolate spatial PFAS contamination hotspots, which is critical for identifying communities that are vulnerable to PFAS exposure.

## 2. Data

County population, demographic, income, poverty, and housing data are collected from the American Community Survey 5-year estimates [29–32]. Per capita income is adjusted for purchasing power using regional price parities index [33, 34]. Poverty percentage is the share of poor residents relative to total county population. A household is poor if family size adjusted income falls below the threshold set by the US Census Bureau [35]. For instance, if a household consists of one person under 65 years of age and their annual income is less than $14,097, then they are considered below the poverty line. If the household consists of three people, the threshold increases to $21,559.

Non-white population is the difference between county total and white population. Number of non-white poor individuals is estimated by subtracting the number of white poor individuals from the total number of poor individuals. Finally, non-white poverty rate is the share of non-white poor individuals relative to total non-white population. On average, 23% of county population is non-white, ranging from 1% to 84%. Overall poverty is 16% (4–44%), with 13% (3 to 39%) for white population and 24% (1–84%) for nonwhite population (Table 1). The housing density is calculated by dividing the total number of housing units in the county by the total area of the county. The average number of houses per square mile is 286, with a range from 0.5 to 4832.

**Table 1:** Description and summary statistics of the variable used in the regression analysis.

Variables	Description	Mean	SD	Min	Max
Dependent Variables					
Concentration (mug/L)					
PFBS	Perfluorobutane sulfonic acid	0.0001	0.0044	0	0.3700
PFHpA	Perfluoroheptanoic acid	0.0001	0.0022	0	0.0869
PFHxS	Perfluorohexanesulfonic acid	0.0007	0.0137	0	0.7300
PFNA	Perfluorononanoic acid	0.0000	0.0009	0	0.0559
PFOA	Perfluorooctanoic acid	0.0004	0.0058	0	0.3490
PFOS	Perfluorooctanesulfonic acid	0.0010	0.0209	0	1.8000
PFAS	Sum of concentration of all above six PFAS tested	0.0024	0.0381	0	2.7000
Binary indicator					
PFBS	1 if water system has PFBS in at least one sample	0.0005	0.0219	0	1
PFHpA	1 if water system has PFHpA in at least one sample	0.0064	0.0798	0	1
PFHxS	1 if water system has PFHxS in at least one sample	0.0054	0.0731	0	1
PFNA	1 if water system has PFNA in at least one sample	0.0005	0.0231	0	1
PFOA	1 if water system has PFOA in at least one sample	0.0106	0.1024	0	1
PFOS	1 if water system has PFOS in at least one sample	0.0077	0.0876	0	1
PFAS	1 if water system has PFNA in at least one sample	0.0162	0.1264	0	1
Independent Indicator Variables					
Binary PWS Characteristics					
PWS size: Small	1 if water system has 10000 or less consumers	0.0894	0.2853	0	1
Water Source: SW	1 if water source is surface water	0.3549	0.4785	0	1
Water Source: MX	1 if water source is mixed water	0.0226	0.1488	0	1
Water Source: SIG	1 if water source is ground, but influenced by surface water	0.0121	0.1095	0	1
Population (1,000)	Population of county in which PWS is located	970.04	1786.04	1.97	10,100
Non-White Population	% of other than white people in the county	23.57	13.89	0.98	83.62
Total Poverty	% of the poor people	15.50	5.88	3.63	43.94
White Poverty	% of white population who are poor	12.98	5.21	2.91	39.37
Non-white Poverty	% of non-white population who are poor	23.57	13.89	0.98	83.62
Housing density	Number of housing unit per square mile	286.49	392.18	0.50	4832.06
Contribution (%) to the GDP from					
Agriculture		2.2	5.1	0	51.6
Durable goods manufacturing		7.2	6.5	0	56.7
Non-durable good manufacture		5.9	6.9	0	94.6
Healthcare and social assistance		7.7	3.3	0	41.9
Food and accommodation		2.9	1.9	0.0	33.5
Government enterprise		14.02	7.7	1.1	75.3

The county Gross Domestic Product (GDP) and shares of GDP from Agriculture, Forestry, Fishing and Hunting; non-durable and durable goods manufacturing; Health Care and Social Assistance; Accommodation and Food Services; and government enterprise are obtained from the U.S. Bureau of Economic Analysis [36]. On average, Government enterprise has the highest contribution to the county GDP (14%) followed by health care (8%), durable goods manufacturing (7%), non-durable goods manufacturing (6%), food and accommodation (3%), and agriculture (2%) (Table 1).

National PFAS contamination data are from the US EPA’s National Contaminant Occurrence Database (NCOD), which provides UCMR3 data from 2013 to 2016 [37]. UCMR3 is the most comprehensive and consistent national data source on PFAS contamination in Public Water Systems (PWS). We focus on the 48 lower US states, excluding Washington DC. In the lower USA, total of 35,589 water samples were collected from 1,616 counties, 4,782 PWSs, and 14,607 water supply facilities at the entry point to the distribution system. The selected PWSs were tested quarterly or bi-annually for a year depending on the intake water source [38]. PWSs that rely on Ground Water (GW) were sampled twice, with a 5- or 7-month interval, while those that use Surface Water (SW), Mixed Water (MX), or ground water that is “under the direct influence of surface water” (SIG) were sampled four times, once in each consecutive quarter. Multiple water supply facilities may be part of each PWS, and water samples were collected from each facility of the selected PWSs. The collected samples were analyzed at an EPA-approved laboratory. UCMR3 database includes detailed information on the PWSs’ characteristics, including county, zip code, PWS ID/name, Water Supply Facility ID/name, size, water source, sample point name, sample ID, sampling date, and analytical results for six types of PFAS.

**Fig 1:**
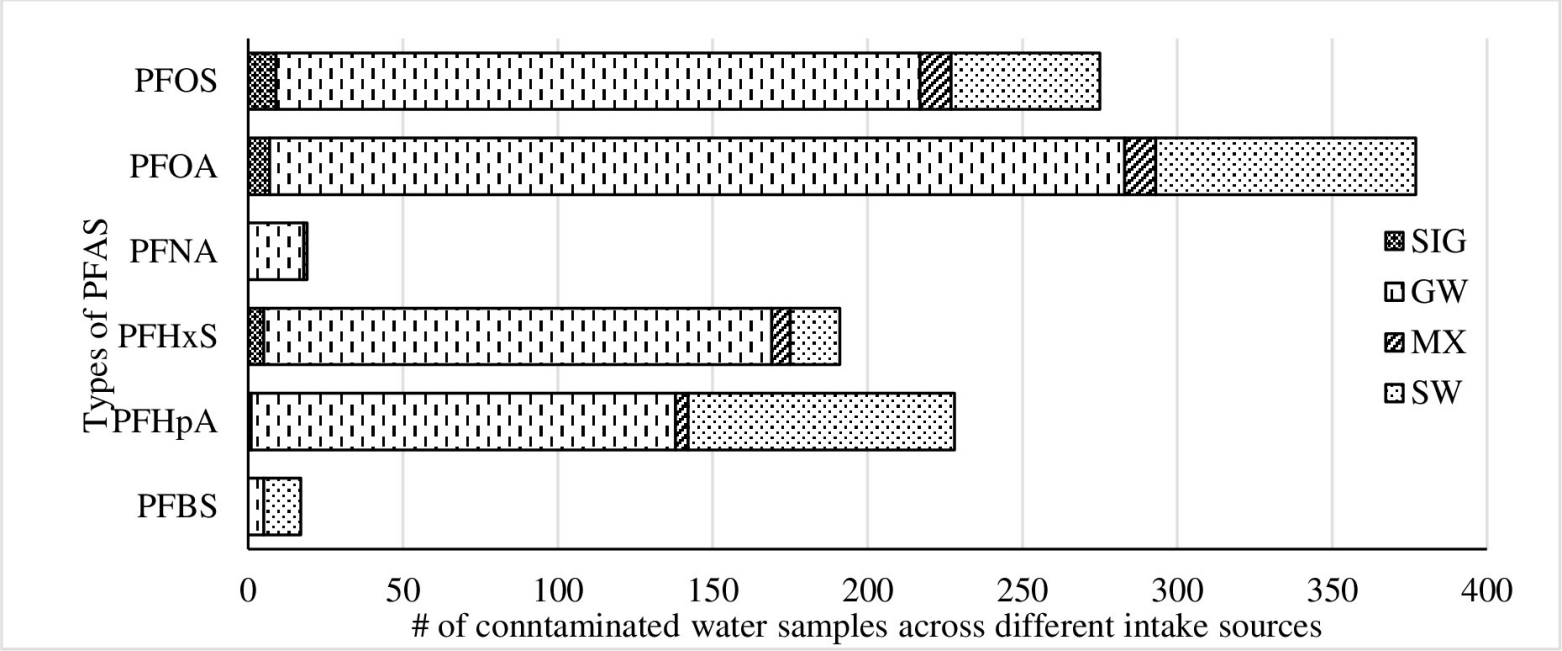
Contaminated samples by water source.

At least one of the six tested PFAS was detected in 33 states (68.7%), 121 counties (7.49%), 193 PWS (4.04%), and 345 PWS-Facilities (2.36%). Overall, 1107 water samples were contaminated, which is 0.52% of the sample (Fig 1). The most contaminations were observed for PFOA (n = 377) followed by PFOS (n = 275), PFHpA (n = 228), PFHxS (n = 191), PFNA (n = 19), and PFBS (n = 17). PFOA, PFOS, PFHpA, PFHpX, PFNA and PFBS were detected in 27, 24, 22, 22, 4 and 7 states, respectively affecting around 16 million people (5.07% of the contiguous US population). The UCMR3 summary statistics of PFAS in the lower USA are presented in S1 Table. Although the recent EPA Health Advisory included PFOA, PFOS, PFBS, and GenX (Hexafluoropropylene Oxide Dimer Acid and its Ammonium Salt), this study does not examine PFBS and GenX as UCMR3 did not have enough positive data on PFBS and did not collect any data on GenX.

Public Water Systems (PWS) can have multiple water supply facilities, each with separate water intake. In the lower USA, there are 14,706 water supply facilities and 4,782 PWS. Of these facilities 22% rely on surface water (SW), 75.8% on groundwater (GW), 1.4% on mixed water (MX), and 0.8% on surface water influenced groundwater (SIG). The UCMR3 data show that PFOA, PFOS, PFHxS, and PFNA contamination is higher if the water source includes groundwater (i.e., GW, MX, or SIG) (Fig 1 and S2 Table), which is consistent with prior literature [8, 26].

**Fig 2:**
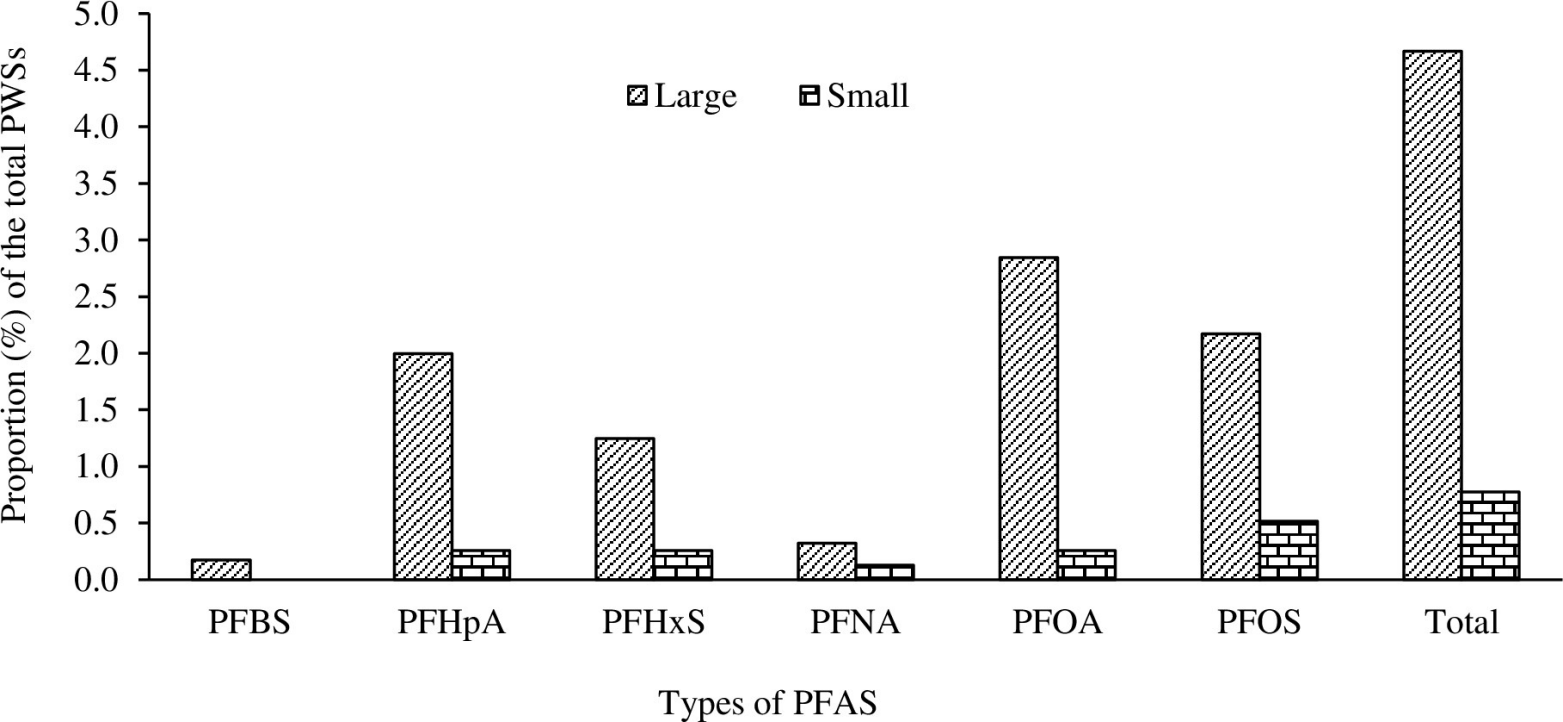
Proportion of the contaminated large and small PWSs.

PFAS contamination differs depending on the size of PWSs (Fig 2). PWSs that serve at least 10,000 people are more likely to be contaminated than smaller PWSs. Specifically, 4.7% of large PWSs showed contamination with at least one PFAS, as opposed to the 0.8% of small PWSs (S2 Table). These findings corroborate the results of Guelfo and Adamson [26] and underscore the importance of PWS size in assessing the vulnerability to PFAS contamination.

**Fig 3:**
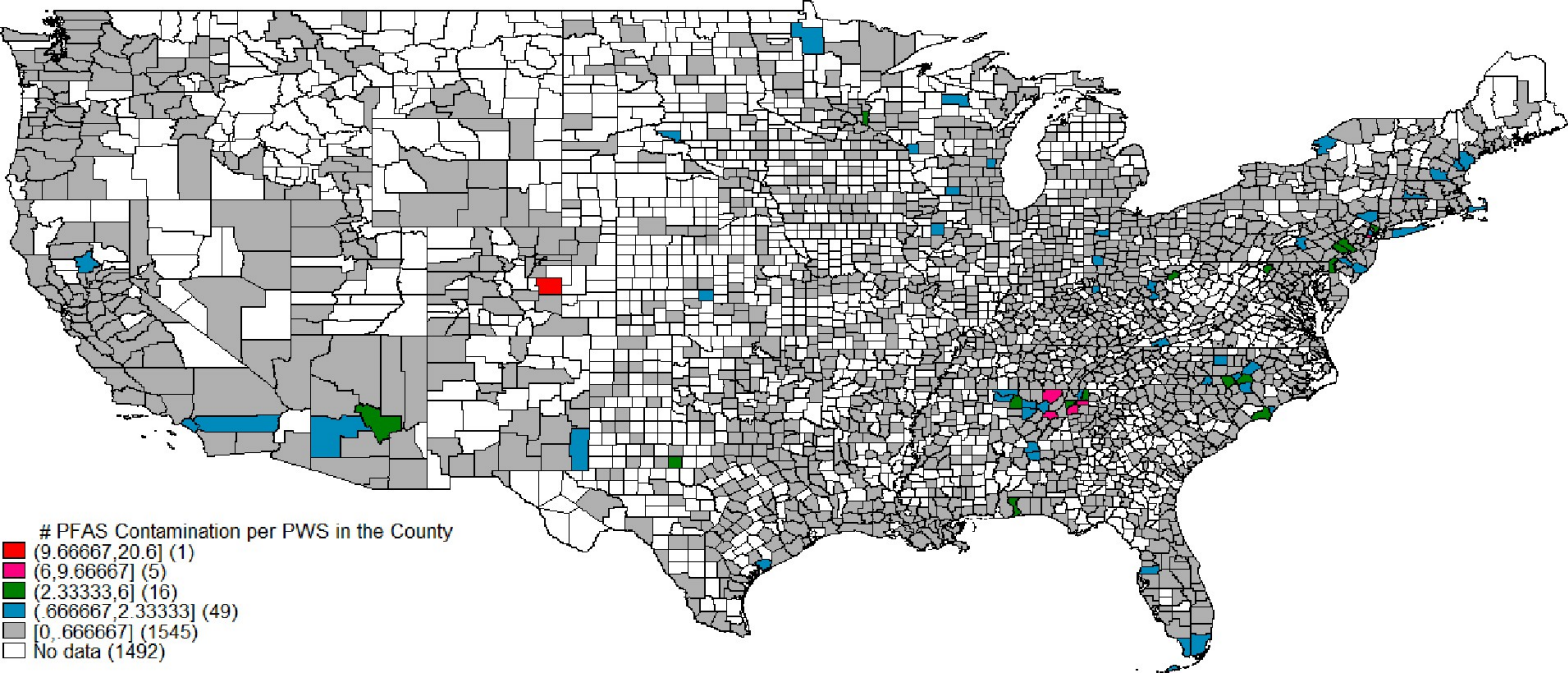
Number of PFAS contaminated water samples per PWS and county.

To provide a visual representation of spatial PFAS contamination distribution, we show cumulative county scale results across six PFAS per PWS in Fig 3. The aggregate number of positive samples in each county is divided by the number of PWS in each county. We used the Jenks natural breaks classification method [39] to classify counties into groups based on contamination per PWS and county. Fig 3 shows that PFAS contaminations are more prevalent in Eastern than in western US counties. Some of the most contaminated counties are in Colorado, Alabama, Georgia, Delaware, New Jersey, and North Carolina. S1 Fig shows contamination per PWS in each county for PFOA, PFOS, PFHpA, and PFHxS individually.

Several data limitations should be acknowledged. First, socio-demographic variables were obtained from the US Community Survey 5-year estimate, which represent weighted five-year averages. Hence, averages, rather than particular year statistics were used for race, housing density, and poverty. Second, GDP contributions from various sectors are highly aggregated. This limitation prevents a more specific analysis of the industrial sources of PFAS contamination. Third, UCMR includes data from all large facilities, but only a random subsample of small facilities that serve 10,000 people or less. If the EPA’s randomization strategy was effective, then this asymmetry should not affect the results. Fourth, we do not examine the effects of natural external factors like climate or geology due to data limitations. Depending on data availability in the future, one could examine the impacts of more intense and frequent precipitation, floods, and heatwaves on PFAS in drinking water.

## 3. Methods

### 3.1 Hot spot analysis

Spatial autocorrelation is used to identify county-scale hot spots for PFOA, PFOS, PFHpA, PFHxS individually and all PFAS cumulatively [40–43]. Hot spot analysis is not performed for PFNA and PFBS due to limited positive samples. Getis-Ord ($G_{i}^{*}(d))$ z- statistic is used to identify the spatial clusters, where a county is in a statistically significant hot spot if it has sufficiently high contamination and is surrounded by other contaminated counties. Higher z-values indicate more intense clustering. Counties with z-value at or above 2.58 and 1.96 are in hot spots at 1%, and 5% level of significance, respectively. Following Getis and Ord [41] and Ord and Getis [43] the Getis-Ord $G_{i}^{*}(d)$ statistic for PFAS contamination per PWS in county *i* is estimated as

$$G_{i}^{*}(d)=\left(\sum_{j=1}^{N}w_{ij}(d)x_{j}\right)/\left(\sum_{j=1}^{N}x_{j}\right)$$ (1)

where, *x*_*j*_ is number of contaminated samples per PWS in county *i*, *N* is the number of counties, and *w*_*ij*_(*d*) is 1 if bilateral distance between county *i* and county *j* is within the threshold distance *d* and 0 otherwise. We use d = 146.2 km threshold distance following Allaire et al. [40], such that each county has at least one neighbor.

The standardized Getis-Ord $G_{i}^{*}(d)$ statistic, or the z-value, is computed as follows using nonbinary spatial weight matrices *w*_*ij*_ [42]:

$$Standardized\;G_{i}^{*}(d)=\left(G_{i}^{*}(d)-E\{G_{i}^{*}(d)\}\right)/\left(S\sqrt{Var\{G_{i}^{*}(d)\}}\right)$$ (2)

where,

$$E\{G_{i}^{*}(d)\}=\left(\sum_{j=1}^{N}w_{ij}(d)\right)/N$$ (3)

$$Var\{G_{i}^{*}(d)\}=(\left(N\sum_{j=1}^{N}w_{ij}^{2}(d)-{\left\{\sum_{j=1}^{N}w_{ij}^{2}(d)\right\}}^{2}\right)/N^{2}(N-1)){\left(s/\bar{x}\right)}^{2}$$ (4)

Sample mean ($\bar{x}$) and sample variance (*s*^2^) in Eq (4) are defined as follows:

$$\bar{x}=\left(\sum_{j=1}^{n}x_{j}\right)/N$$ (5)

$$s^{2}=\left(\sum_{j=1}^{n}\left(x_{j}^{2}-{\bar{x}}^{2}\right)\right)/N$$ (6)

### 3.2 Regression analysis

We examine physical, socioeconomic, and industrial characteristics of PWS and surrounding communities that may be associated individual (PFOA, PFOS, PFHpA) and cumulative PFAS (PFOA, PFOS, PFHpA, PFNA, PFBS, and PFHxS) contamination of drinking water using Probit and Tobit models. PFNA, PFBS, and PFHxS contaminations are not examined due to insufficient number of positive samples. Statistically significant correlations between any of the physical, socioeconomic, and industrial characteristics of PWS and the concentration or presence of PFAS in the PWS identify potential reasons for PFAS contamination.

The UCMR3 dataset limitation is in the non-reporting of results below the Minimum Reporting Level (MRL) [25]. Test results below the MRL are reported as zero, implying data censoring. None of the earlier studies explicitly considered this censoring issue. Extending prior literature, we use a Tobit limited dependent variable model to account for censoring in the UCMR3 PFAS data [28, 44].

The drinking water PFAS reported in UCMR3 is a limited dependent variable because PFAS below MRL (Minimum Reporting Level) are not reported [45]. PFAS in UCMR3 are only reported if the concentration exceeds the MRL threshold. Denoting the true PFAS concentration as y*, the EPA-reported PFAS concentration is y = y*, if y* ≥ MRL. If the concentration is positive but less than the MRL, then y* is not observed, and y is set to 0.

$$y_{it}=\left\{\begin{array}{cc} y_{it}^{*}\;if & y_{it}^{*}\geq MRL\\ 0\;if & y_{it}^{*}<MRL \end{array}\right\}$$

where, MRL is a constant and $y_{i}^{*}$ is missing if it is less than MRL.

Since positive values between 0 and the MRL are reported as 0, the censored mean of y lags behind the mean of y* [45]. Censoring also results in a distribution of y that is different from the true population distribution. Ordinary Least Squares (OLS) regression in this case produces biased and inconsistent parameter estimates [44]. To address the inconsistency and bias in estimation, we use a Tobit model. The Tobit model directly models the observed values (y) and incorporates the probability of observing censored data.

The Tobit model produces unbiased and consistent estimations by maximizing the censored log-likelihood function, which is a mixture of discrete and continuous densities. The continuous density represents the density when y* ≥ MRL, while the discrete density function represents the density of a binary outcome for when y* ≥ MRL versus when y* < MRL [45].

Since Ordinary Least Squares (OLS) regression produces biased results in the presence of data censoring, we do not present the OLS results but have those, along with Heckman two step model results, available upon request.

The left-censored Tobit Model [28, 45, 46] is formulated as follows:

$$y_{it}^{*}=\beta_{0}+\beta_{j}C_{itj}+\beta_{k}E_{itk}+{\beta_{l}X}_{il}+\beta_{m}S_{m}+\beta_{t}T_{t}+\mu_{it}$$ (7)

where, $y_{it}^{*}$ is the latent variable for PFAS concentration in the PWS facility *i (i = 1*:*n)* in the year *t*. ***C***_***i****tj*_ is the nt*j matrix of socioeconomic characteristics j (j = population, nonwhite population, poverty (or income), nonwhite poverty, and housing density) in year t of the county where PWS facility *i* is located. We run two regression models, one with poverty and the other with income as independent variables. ***E***_***itk***_ is the nt*k matrix of GDP share from sector *k* (k = Agriculture, forestry and fisheries; durable goods manufacturing, non-durable goods manufacturing; health care and social assistance; food and accommodation; government enterprise) in year t in the county where PWS facility *i* is located. ***X***_***il***_ is the matrix of PWS characteristics *l* (*l* = size of the PWS and source of the water). Binary state (*S*_*m*_), and year (*T*_*t*_) indicators are included to account for time invariant state factors and state invariant year factors. Population and housing density data are log transformed.

We also use Probit regression to examine binary contamination occurrence (likelihood of contamination) as follows,

$${Pr(y}_{it}=1|X)=\Phi (\beta_{0}+\beta_{j}C_{itj}+\beta_{k}E_{itk}+{\beta_{l}X}_{il}+\beta_{m}S_{im}+\beta_{t}T_{t})$$ (8)

where, Pr(*y*_*it*_ = 1|*X*) is the probability of observing a PFAS-positive sample for i^th^ PWS in year t, Φ is a cumulative density function of normal distribution, and the rest of the notation is consistent with Eq (7).

## 4. Results

### 4.1 Contamination hot spots

**Fig 4:**
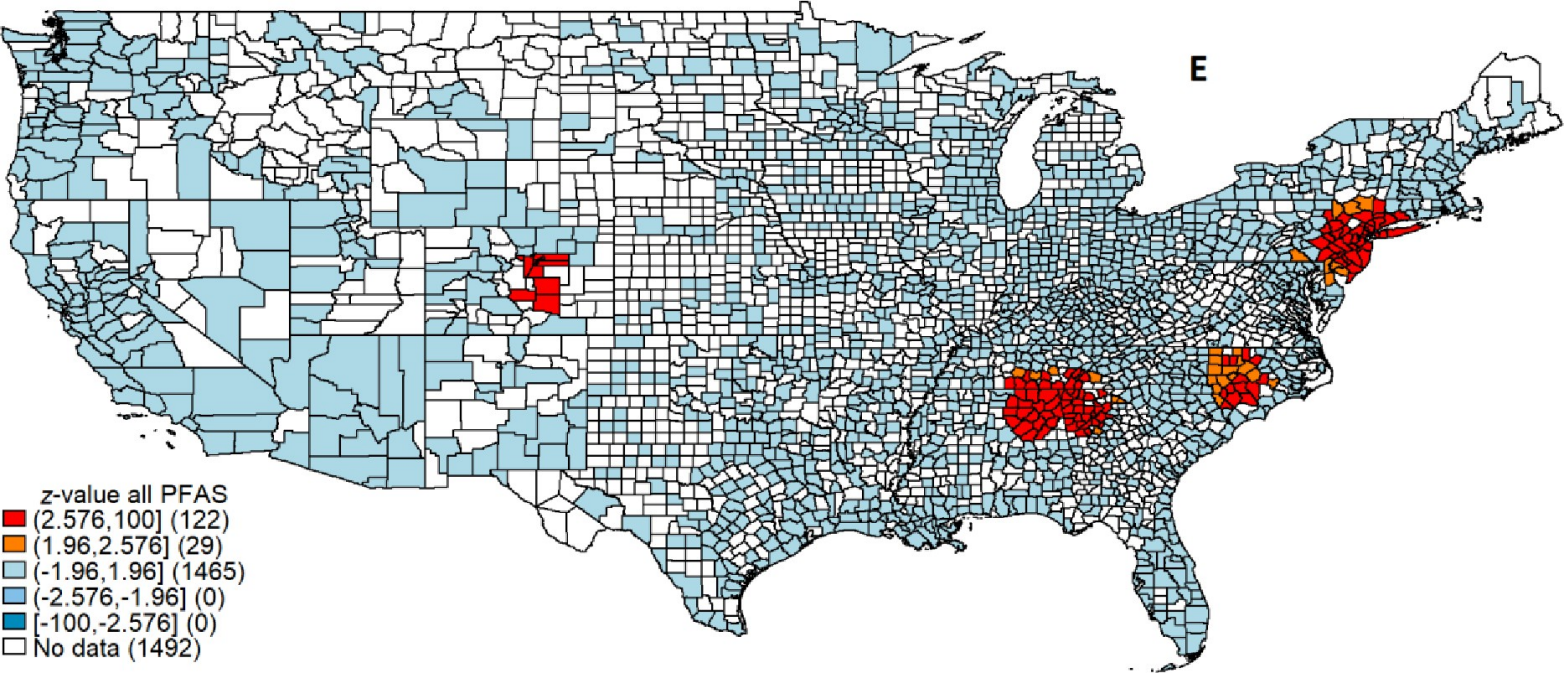
PFAS contamination hot spots. Note: Legend intervals are based on z-values from standardized Getis Ord statistics. Counties with z-values ranging between 1.96 and 2.576 indicate hot spots at a 5% significance level, while z-values of 2.576 and higher indicate hot spots at 1% significance level. The values in the first set of parentheses indicate the z-value ranges and the values in second set of parentheses indicate the number of counties in the group.

We identified four cumulative PFAS contamination hot spots (Fig 4), which include 10 states and 149 counties. The hotspot with the greatest number of counties spans across Alabama (18 counties), Georgia (31 counties), and Tennessee (17 counties). The second largest hot spot spans New Jersey (20 counties), Pennsylvania (14 counties), New York (10 counties), Delaware (2 counties) and Connecticut (2 counties). The third largest hotspot is on the border of North Carolina (25 counties) and South Carolina (2 counties). The smallest of the four hotspots is in Colorado (10 counties). We also identified PFOA, PFOS, PFHpA, and PFHxS hot spots individually (S2 Fig). A list of states and counties in the hot spots, along with the z-scores are reported in S4–S8 Tables.

The regional hot spots have PFAS manufacturing plants, industrial sites that utilize PFAS, and/or densely populated communities [47–52]. For instance, the hotspot in the northeastern US (NY, DE, NJ, PA, and CT) is in a densely populated region with high consumption of household goods containing PFAS, such as detergents, cosmetics, and food packaging [9]. This hot spot is home to 25 million people. The average population density in this region is 937 people per square mile, which is nine times higher than the national average. This hot spot contains the nation’s top three most densely populated counties and five out of ten most densely populated counties in the US. Consumption of PFAS-containing products results in the release of these compounds into surface and groundwater through wastewater systems that lack the capacity to remove PFAS. Subsequently, drinking water can be contaminated if community water systems (CWSs) that lack PFAS treatment capacity draw from contaminated intakes.

The Colorado hotspot encompasses counties with the US Space Command, an Air Force Base, and an Air Force Academy which use PFAS as part of their operations. Aquifer in this region is contaminated by Aqueous Film Forming Foam (AFFF) use at the Air Force Base. Quezada Davalos et al. [51] found that sites near AFFF use have significantly higher PFAS concentrations than distant sites.

The hot spot in Georgia, Tennessee, and Alabama may be attributed to the use of PFAS in industrial production. de Amorim et al. [48] report that the manufacturing industry and the Air Force Base in Georgia are major sources of PFAS contamination in drinking water. PFAS chemicals are extensively used in carpet production and firefighting foam. PFAS contamination from the carpet industry in this hotspot was also documented by Viticoski et al. [53]. Slightly elevated levels of PFAS were also observed in the rivers downstream from cities and landfills, which points to household consumption as a source of PFAS.

Prominent manufacturers like Dupont and 3M located in this region have faced legal challenges from cities, counties, and states. Some cases have been resolved with significant settlements, while others are still ongoing. For example, the responsible industries settled a lawsuit in the Tennessee River region of northern Alabama by paying $98 million for contaminating the river and downstream drinking water [47]. They also paid $35 million to the West Morgan-East Lawrence Water Authority in Alabama. Additional lawsuit settlement involved $100 million paid to the affected community [54]. In 2023, the Tennessee attorney general filed another lawsuit against more than 20 PFAS manufacturers, alleging environmental contamination and harm to natural resources and public health [55].

The hotspot in North and South Carolina is associated with the manufacturing, consumption, and industrial use of PFAS [49, 50, 52]. Ehsan et al. [49] discussed manufacturing sites, firefighting foam, waste disposal and treatment plants, landfill leachate, and industrial emissions as sources of PFAS contamination in drinking water. PFAS can enter the wastewater treatment system or end up in landfills through waste management, ultimately contaminating the drinking water source. Scruggs [52] traced the roots of PFAS in this hotspot back to 1980 when DuPont’s Fayetteville Works plant initiated PFAS production. The contamination of the Cape Fear River watershed can be directly attributed to the PFAS manufacturing activities of DuPont’s Fayetteville Works plant.

### 4.2 Regression results

The cumulative PFAS results from the Probit and Left Censored Tobit models are presented in Table 2. Tobit model shows correlations between cumulative PFAS (sum of PFOS, PFOA, PFHxS, PFNA, PFBS and PFHpA) and the corresponding independent variables, while the Probit model examines the likelihood of a PWS showing a positive PFAS sample. The results show that the Tobit model provides a better fit than Probit based on Akaike’s Information Criteria (AIC) and Bayesian Information Criteria (BIC) values. Nevertheless, although the results are qualitatively consistent, we present both model results because they have technically different interpretations. We use poverty and income in separate models to control the effects of affluence on PFAS occurrence. The specifications with poverty and income produce similar results, and we focus the discussion on the model with poverty. The corresponding PFOA, PFOS, and PFHpA results are provided in the S9 and S10 Tables.

**Table 2:** Tobit and Probit results for cumulative PFAS contamination.

	Tobit		Probit	
	Model 1	Model 2	Model 1	Model 2
	Marginal effect		Marginal Effect at means	
PWS size (1 = small, 0 = large)	-0.00014550*** (0.00002710)	-1E-04*** (0.000027)	-0.0070471*** (0.000989)	-0.0070*** (0.0010)
Water source (SW = 1)	-0.00016320*** (0.00003360)	-2E-04*** (0.000034)	-0.0060083*** (0.0010733)	-0.0062*** (0.0011)
Water source (MX = 1)	-0.00005630 (0.00007000)	-6E-05 (0.000071)	-0.0016275 (0.002817)	-0.0017 (0.0028)
Water source (SIG = 1)	0.00009870 (0.00007750)	9E-05 (0.000078)	0.0057402** (0.0030902)	0.0052 (0.0031)
Population (log)	0.00005740*** (0.00001930)	5E-05*** (0.000019)	0.0017164** (0.0007303)	0.0014* (0.0007)
Non-White population (%)	-0.00000626*** (0.00000168)	-7E-06*** (0.000002)	-0.0002507*** (0.0000596)	-0.0003*** (0.0001)
Poverty (%)	-0.00000642 (0.00000545)	-	-0.0002619 (0.0002196)	
Nonwhite Poverty (%)	0.00000265 (0.00000283)	-	0.0001473 (0.0001157)	
Regional Price Parities (RPP) adjusted per capita income (USD/person)	-	-4E-09 (2.71E-09)		-2.26E-07*** (1.08E-07)
Log Housing density (house/sq mil)	0.00008460*** (0.00002150)	1E-04*** (0.000023)	0.0040218*** (0.0007697)	0.0045*** (0.0008)
*Percentage Contribution to the GDP from*				
Agriculture	-0.00001100** (0.00000512)	-1E-05*** (0.000005)	-0.0004947** (0.0002042)	-0.0006*** (0.0002)
Durable goods manufacturing	-0.00000737** (0.00000298)	-8E-06*** (0.000003)	-0.0003328*** (0.0001142)	-0.0004*** (0.0001)
Non-durable good manufacture	0.00001300*** (0.00000249)	1E-05*** (0.000002)	0.0005242*** (0.0000789)	0.0005*** (0.0001)
Healthcare and social assistance	0.00001020** (0.00000403)	8E-06** (0.000004)	0.0003494 (0.0001585)	0.0002*** (0.0002)
Food and accommodation	0.00000783 (0.00000546)	7E-06 (0.000005)	0.0002766 (0.0002204)	0.0003*** (0.0002)
Government enterprise	0.00000738*** (0.00000188)	6E-06*** (0.000002)	0.0002577 (0.0000694)	0.0002*** (0.0001)
Observations	35,589	35,589	30,777	30,777
Prob > chi2	0	0.00000	0	0.00000
Pseudo R2	0.2	0.2001	0.1413	0.1418
AIC	4093.793	4091.17	5029.111	5024.347
BIC	4526.262	4515.16	5445.837	5432.738

Tobit outputs are for the marginal effect of the censored sample.

Standard errors in parentheses

*** p<0.01

** p<0.05

* p<0.1

#### 4.2.1. PWS characteristics

PFAS contamination is less likely in small PWSs. The Tobit model indicates that on average, small PWSs have 0.145 ng/L (0.000145μg/L*1000 = 0.145 ng/L) less PFAS than large PWSs. Similar results are observed from the probit model, which shows that smaller facilities are less likely to have PFAS. These results are consistent with previous PFAS studies, which show greater susceptibility of larger PWSs [8, 26]. The result is also consistent with Rahman et al. [56], who found more SDWA-regulated contaminant violations by large PWSs than by small ones in Arizona. However, several SDWA compliance studies find that small PWSs have more SDWA violations [40, 57, 58].

PWSs that rely on surface water sources are less likely to experience PFAS contamination than to those that depend on groundwater. PWSs that use surface water have 0.163 ng/L less PFAS concentration than groundwater PWSs. This finding is in line with other PFAS contamination studies [8, 26]. These results are in contrast with the SDWA-regulated contaminants, which are more prevalent in PWSs that rely on surface water sources [40]. Hence, surface water may pose a greater risk for some of the SDWA-regulated contaminants, while groundwater may pose a greater risk in terms of PFAS contamination.

The documented discrepancy in PWS susceptibility across SDWA-regulated contaminants and PFAS sheds light on biophysical differences between these pollutants. The lower susceptibility of groundwater than surface water dependent PWSs suggests that aquifer percolation acts as a filtration mechanism for SDWA regulated pollutants. In contrast, these filtration processes appear to be ineffective against PFAS. The greater presence of PFAS in PWSs that rely on groundwater indicates that these persistent compounds resist degradation through natural percolation processes that help filter SDWA-regulated pollutants [59, 60]. Instead, PFAS compounds persist and accumulate, posing a concern for the quality and safety of groundwater resources [59].

#### 4.2.2. Socioeconomic characteristics

PFAS contamination is more likely in PWSs in areas with greater population densities. For every 1% increase in population, PFAS concentration increases by 0.00057 ng/L (0.00005740μg/L*1000 = 0.0574 ng/L). Similarly, PFAS increases by 0.00085 ng/L for every 1% increase in housing density. The positive effect of population and housing density may be associated with greater consumption of goods that contain PFAS, including detergents, cleaning supplies, cosmetics, carpeting, clothes, and others. PFAS from these products wind up in sewage systems and eventually drain into water bodies that serve as intake sources for PWSs. If PWSs and wastewater treatment systems lack the capacity to filter PFAS, drinking water in areas with greater population and housing density can contain elevated levels of PFAS.

PFAS contamination is negatively correlated with the non-white population. We find that for every 1% increase in the non-white population, the concentration of PFAS decreases by 0.0063 ng/L. Poverty has no impact on PFAS concentration, while per capita income has a negative effect on the probability of experiencing PFAS contamination. These findings are in contrast with what is observed in the case of SDWA-regulated contaminant violations, which are more prevalent in disadvantaged communities and in areas with higher poverty [40, 61–63]. PWSs in disadvantaged communities have limited access to financial and other resources, resulting in poorer infrastructure and maintenance, which contributes to water quality violations [61]. In the case of PFAS contamination, however, lack of financial resources does not seem to be associated with contamination. One possible explanation for this outcome is that during the UCMR3 program time frame, PFAS in drinking water and the resulting health risks were not widely publicized. As a result, mitigation efforts were not implemented even in wealthier communities. The lack of awareness regarding the presence of PFAS in drinking water, the adverse health impact of these chemicals, and the lack of efficient filtration technology have likely contributed to statistically insignificant differences across poverty delineation.

#### 4.2.3. Industrial composition

PWSs in areas where agriculture, forestry, and fishery sectors represent a significant share of the local economy are less prone to contamination. A 1% increase in the share of agriculture, forestry, and fishery in the county GDP results in a 0.01 ng/L decline in PFAS concentration. Similarly, the likelihood of PFAS detection above the UCMR MRL decreases by 2% as the share of agriculture and forestry GDP increases by 1%. These results are expected because there is no evidence of a significant PFAS use as input in agricultural production besides the use of municipal biosolids as fertilizer [64–66]. However, our results suggest that this practice does not generally produce significant PFAS contamination nationally. Counties dominated by agricultural industry are also less densely populated, which results in lower consumption of products with PFAS and less leaching into ground and surface waters.

The correlation between county GDP from the non-durable goods manufacturing industry and PFAS contamination in local PWSs is positive and statistically significant. A1% increase in the share of GDP from non-durable goods manufacturing leads to a 0.01 ng/L increase in drinking water PFAS concentration. Similarly, the likelihood of PFAS contamination increases by 2.2% for a 1% increase in the share of county GDP coming from the non-durable goods manufacturing industry. Non-durable goods, which have an average life of less than three years, include a wide range of products such as textiles, food packaging material, clothing, cosmetics, hygiene products and more [67]. Manufacturing of these goods often involves the use of various PFAS compounds, which may explain the positive correlation between elevated contamination and the prominence of non-durable goods manufacturing industry in the local economy.

The healthcare and social assistance industry also exhibits a notable positive correlation with PFAS contamination. With 1% increase in the healthcare and social assistance industry’s share to the county GDP, PFAS concentration increases by 0.01 ng/L and the probability of contamination increased by 1.2%. PFAS materials are used in these establishments, instruments, and products due to their resistance to heat, water, and chemical degradation. These properties are critical for hospital operations [68]. PFAS are commonly used in medical implants and devices such as vascular grafts, surgical meshes, gowns, catheter tubes, filters, needle retrieval systems, tracheostomies, inhalers, catheter guide wires, and imaging products [69]. The consistent positive correlation between PFAS concentration and the proportion of the health sector in the local economy suggests that the healthcare industry may serve as a significant source of PFAS in the environment.

We observe that PFAS contamination is more likely for PWSs located in counties with more government enterprises. For every 1% increase in the government enterprises’ share of county GDP, PFAS concentration increases by 0.0073 ng/L and the probability that the PWS has PFAS contamination increases by 1.2%. Government enterprises such as military, firefighting, and government-operated airports use firefighting foams and heat-resistant equipment that contain PFAS [8].

## 5. Discussion

Understanding the socio-economic and industrial factors associated with drinking water PFAS contamination is critical for developing effective mitigation strategies. Hot spot analysis identifying regional contamination clusters helps pinpoint vulnerable communities and prioritize mitigation efforts to contain and eliminate PFAS from the drinking water systems. In this study, hot spots of PFAS in drinking water are detected in the southeastern US, including—Alabama, Georgia, and North Carolina; the western US in Colorado; and the northeast US in New Jersey, New York, and Connecticut.

A higher concentration of PFAS in larger PWS is consistent with previous PFAS research [8, 26]. PFAS contamination differs from the regulated SDWA pollutant violations, which are more common in smaller PWS [40, 58, 63, 70]. One reason for this difference may be that smaller PWSs are generally in smaller communities, which consume fewer goods with PFAS. Hence, intake of source water by smaller PWSs may be less contaminated with PFAS than intake of larger PWSs. Additionally, the superior credit ratings and financial strength of larger PWSs do not seem to affect PFAS contamination because at the time of UCMR3 data collection health risks of PFAS in drinking water were not widely understood and removal of PFAS was not yet a priority.

We find that PWSs that rely on groundwater experience greater PFAS contamination. This result is consistent with prior PWS-PFAS contamination studies [8, 26]. The PFAS susceptibility of PWSs that rely on groundwater is in contrast with the SWDA-regulated contaminants [40, 71]. Allaire et al. [40], for instance, show that violations of drinking water quality regulations are significantly higher for PWSs that rely on surface water. The advantage of groundwater as an intake source for PWSs is that aquifers provide natural filtration before water is pumped for treatment and delivery. These filtration processes remove many of the SDWA-regulated contaminants. However, PFAS do not biodegrade and are much more persistent [59, 60]. As a result, natural groundwater filtration is less effective for removing these compounds than SDWA-regulated pollutants. Moreover, the persistence of PFAS implies that these compounds can bioaccumulate in aquifers over time and can continue to affect aquifer drinking water supplies as legacy pollutants. Hence, unlike SDWA-regulated pollutants, a particular focus on aquifer sources may be justified for remediating PFAS in drinking water.

PFAS concentration is greater in PWSs in more densely populated counties. This result is likely due to the greater consumption of PFAS-containing goods in densely populated regions [9]. PFAS from the use of household products like detergents, cleaning agents, clothing, food packaging, and others propagate to the wastewater treatment systems, which are equipped to remove PFAS prior to discharge. Consequently, raw drinking water sources in more densely populated areas are more contaminated [72]. Since some drinking water treatment facilities also lack the capability to remove PFAS from drinking water, more PFAS contaminated intake implies more PFAS in finished drinking water. Hence, unless appropriate technology is deployed to remove PFAS from treated drinking water and/or from treated wastewater discharge, mitigation of PFAS in drinking water will require addressing PFAS leakage from household consumption. This structural relationship points to PFAS contamination as an externality from the consumption of household products that contain these compounds, suggesting a potential role for consumer demand-based instruments as part of the PFAS externality corrective policy.

In general, larger PWSs in wealthier counties have better credit ratings, financial resources and technical capabilities [73, 74]. However, we do not observe a consistent and significant relationship between PFAS contamination and poverty or per capita income in the surrounding community. We also do not find a statistically significant effect of non-white poverty in contrast to studies that document disproportionate SDWA violations in disadvantaged communities that often lack access to policymaking processes or funding [40, 61, 62, 74]. Our results show that limited access to resources is not a significant factor for drinking water PFAS contamination in the UCMR3 data. Instead, our results show that communities with a higher proportion of non-white population experience statistically less PFAS in drinking water. PFAS in drinking water does not depend on community wealth and minority communities are not at a disadvantage. One reason for this result may be that when UCMR 3 data were collected, health effects of PFAS in the drinking and the extent of PFAS contamination in PWSs were not yet documented or publicized. As a result, treatment technologies were not deployed even in the wealthier communities.

PFAS in drinking water also depends on regional industrial composition. The observed negative correlation between PFAS in drinking water and the agriculture, forestry, and fishery sector may be due to the limited use of PFAS inputs in these industries. Counties where these industries represent a significant share of the local economy also tend to be more rural with lower population densities [75] and lower consumption of household goods that contribute PFAS to local waterways. On the other hand, a significantly higher concentration of PFAS is observed in counties with non-durable goods manufacturing industry. Non-durable goods, which have an average life of less than three years, include a wide range of products, including textiles, food packaging materials, clothing, cosmetics, hygiene, and more [67, 76]. Non-durable goods manufacturing often involves the use of various PFAS compounds [77], which may explain the observed positive correlation between PFAS contamination and non-durable goods manufacturing.

The healthcare and social assistance industry is also positively associated with PWS PFAS contamination. Many hospital/health care products, such as surgical gowns, drapes, and flooring, contain PFAS. PFAS are also commonly used in medical devices such as vascular grafts, surgical meshes, catheter tubes and others to enhance longevity [69]. As a result, the health industry may be a significant source of PFAS in local waterbodies. We also observe a positive correlation between PFAS and government enterprises, including military, firefighting, and government-operated airports. These government operations often use fire retardants that contain significant quantities of PFAS. This finding is consistent with Hu et al. [8], who detect more PFAS in areas surrounding AFFF-certified airports and military bases.

Pigouvian taxes, regulations and/or information campaigns can be deployed to address drinking water PFAS contamination [78]. On the production side, processes and inputs that generate PFAS leakage to the environment can be regulated or taxed to narrow the discrepancy between private and social marginal production costs. Our results show that non-durable goods manufacturing, the healthcare industry, and the government sector are correlated with drinking water PFAS contamination. Hence, production activities and inputs that result in PFAS leakage in these sectors may be targeted to mitigate PFAS pollution. Additional studies are needed to identify industrial activities and to estimate the magnitudes of taxes and parameters for regulation.

On the consumption side, our results show that drinking water PFAS-contamination is correlated with population density. We observe this result because areas with larger populations are likely to consume more PFAS containing products. Densely populated areas consume greater quantities of goods like textiles, detergents, personal care products, paints, and food packaging materials that contain PFAS. Inadequate post-consumption handling, disposal, and recycling systems result in the propagation of PFAS into the local watershed and sewage systems, which often lack PFAS treatment equipment. Hence, policies that reduce PFAS leakage from household consumption could be worth considering. Pigouvian taxes can be used to a) decrease demand for PFAS containing products and/or b) raise funds for upgrading wastewater treatment facilities. Information campaigns can also raise awareness about PFAS and its impacts on health and environmental quality.

## 6. Conclusions

We analyze the distribution of PFAS across public water systems (PWSs) in the United States, identify various regional hotspots, and explore the potential correlations between elevated PFAS levels in drinking water and socioeconomic, industrial, and water system attributes. Major findings are that PWSs, which are larger, are in densely populated areas, or rely on groundwater as raw water intake source have greater PFAS concentrations. Drinking water PFAS contamination is also correlated with non-durable goods manufacturing, healthcare, and government enterprises. Conversely, we find lower PFAS contamination in communities with more non-white populations and areas with larger agricultural industries.

Cumulatively, this study suggests that PFAS contamination may originate from industrial activities and consumption of household goods. Drinking water PFAS contamination is a negative externality, which can emerge on the production side from inputs that contain PFAS and on the consumption side from usage of household goods that generate PFAS leakage. Previous literature focused on the industrial sources of PFAS and no attention has been paid to the role of population and consumption. We extend previous literature by documenting that PFAS contamination is not only due to industrial pollution, but is also an externality of consumption in populated areas. We observe that greater population, where consumption of products with PFAS is higher, is a significant source of PFAS contamination. This result is significant because it suggests that mitigating PFAS contamination is not just a matter of controlling pollution from industrial sources. PFAS is also an externality from consumption of many household products. The result also has implications for policy beyond the US. For example, as developing countries continue to grow and catch up with the US standards of living, including consumption, an increase in drinking water PFAS contamination can be expected. Observing these results, other countries can benefit from lessons learned in the US and invest in prevention and preparedness systems to reduce exposure to PFAS.

## Supporting information

S1 Table PFAS in the lower US excluding DC. (DOCX)

S2 Table PFAS contamination by large and small PWSs and by intake water source. (DOCX)

S3 Table Getis-Ord (G_i_(d)) statistics for hot-spot analysis. (DOCX)

S4 Table PFOA contamination hotspots. (DOCX)

S5 Table PFOS contamination hotspots. (DOCX)

S6 Table PFHpA contamination hotspots. (DOCX)

S7 Table PFHxS contamination hotspots. (DOCX)

S8 Table PFAS contamination hotspots. (DOCX)

S9 Table Tobit results for individual PFAS. (DOCX)

S10 Table Probit results for individual PFAS. (DOCX)

S1 Fig Number of different types of PFAS contaminated water samples per PWS and county. Panel A, B, C, and D show the number of PFOA, PFOS, PFHpA, and PFHxS contaminated samples, respectively.
(DOCX)

S2 Fig Hot spot of different types of PFAS contaminations. Panel (A) (B) (C), and (D) shows the hotspots of PFOA, PFOS, PFHpA and PFHxS, respectively. Intervals in the legends are selected based on 1% and 5% levels of significance.
(DOCX)

S1 Text (TXT)

S1 Data (XLSX)

S2 Data (XLSX)
