# Supplementary material for: Peril in the Pipeline: Unraveling the threads of PFAS contamination in U.S. drinking water systems
Source: PLoS One. 2024 Apr 4;19(4):e0299789. doi: 10.1371/journal.pone.0299789 (PMC10994316; doi:10.1371/journal.pone.0299789)
Supplement: S1 Table — (DOCX) [file pone.0299789.s001.docx]

S1 Table. PFAS in the lower US excluding DC.

|  | Total Sampled (n) | Detected | | | | | | |
| --- | --- | --- | --- | --- | --- | --- | --- | --- |
|  |  | PFBS | PFHpA | PFHxS | PFNA | PFOA | PFOS | At least one PFAS |
| States (n) | 48 | 4(8.33) | 22(45.83) | 22(45.83) | 7(14.58) | 27(56.25) | 24(50.0) | 33(68.75) |
| Counties (n) | 1616 | 7(0.43) | 61(3.77) | 43(2.66) | 12(0.74) | 78(4.83) | 60(3.71) | 121(7.49) |
| PWSs (n) | 4782 | 7(0.15) | 82(1.71) | 52(1.09) | 14(0.29) | 116(2.43) | 91(1.90) | 193(4.04) |
| Facilities (n) | 14,607 | 9(0.06) | 136(0.93) | 107(0.73) | 14(0.10) | 227(1.55) | 165(1.13) | 345(2.36) |
| Samples (n) | 35,589 | 17(0.05) | 228(0.64) | 191(0.54) | 19(0.05) | 377(1.06) | 275(0.77) | 578(1.62) |
| Population,  10^6^ (n) | 314.38 | 0.28 (0.09) | 8.76 (2.79) | 5.50 (1.75) | 0.52 (0.17) | 7.73 (2.46) | 10.28 (3.27) | 15.95 (5.07) |

*Note: figures in the parenthesis indicate the percentages of the total(n)*
