# Supplementary material for: Peril in the Pipeline: Unraveling the threads of PFAS contamination in U.S. drinking water systems
Source: PLoS One. 2024 Apr 4;19(4):e0299789. doi: 10.1371/journal.pone.0299789 (PMC10994316; doi:10.1371/journal.pone.0299789)
Supplement: S2 Table — (DOCX) [file pone.0299789.s002.docx]

S2 Table. PFAS contamination by large and small PWSs and by intake water source.

| Attributes | Total Count (n) | Contamination (n(%)) | | | | | | |
| --- | --- | --- | --- | --- | --- | --- | --- | --- |
|  |  | PFBS | PFHpA | PFHxS | PFNA | PFOA | PFOS | At least one PFAS |
| PWSs | 4782 | 7 (0.15) | 82(1.71) | 52(1.09) | 14(0.29) | 116(2.43) | 91(1.90) | 193(4.40) |
| Large | 4008 (83.81) | 7(0.17) | 80(2.00)) | 50(1.25) | 13(0.32) | 114(2.84) | 87(2.17) | 187(4.67) |
| Small | 774 (16.19) | 0(0.0) | 2(0.26) | 2(0.26) | 1(0.13) | 2(0.26) | 4(0.52) | 6(0.78) |
| Source of water to the Water supply facility | | | | | | | | |
| Facility | 14607 | 9(0.06) | 136 ((0.93) | 107(0.73) | 14(0.10) | 227(1.55) | 165 (1.13) | 345(2.36) |
| SW | 3213(22.0) | 5(0.16) | 44(1.37) | 7(0.22) | 1(0.03) | 46(1.43) | 27(0.84) | 74(2.30) |
| GW | 11074(75.8) | 4(0.04) | 88(0.79) | 95(0.86) | 13(0.12) | 173(1.56) | 130(1.17) | 258(2.33) |
| MX | 210(1.4) | 0(0.00) | 3(1.44) | 2(0.95) | 0(0.00) | 5(2.38) | 5(2.38) | 6(2.86) |
| SIG | 110(0.8) | 0(0.00) | 1(0.91) | 3(2.73) | 0(0.00) | 3(2.73) | 4(3.64) | 7(6.36) |

*Note: figures in the parenthesis indicate the percentages of the total(n)*
