# Supplementary material for: Peril in the Pipeline: Unraveling the threads of PFAS contamination in U.S. drinking water systems
Source: PLoS One. 2024 Apr 4;19(4):e0299789. doi: 10.1371/journal.pone.0299789 (PMC10994316; doi:10.1371/journal.pone.0299789)
Supplement: S3 Table — (DOCX) [file pone.0299789.s003.docx]

S3 Table. Getis-Ord (G_i_(d)) Statistics for Hot-Spot analysis.

| Variables | z≤-2.58 | -2.58<z≤ -1.96 | -1.96<z<1.96 | 1.96≤z<2.58 | 2.58≤z |
| --- | --- | --- | --- | --- | --- |
| Total PFOA contamination per PWS in the county | 0 | 0 | 1481 | 19 | 116 |
| Total PFOS contamination per PWS in the county | 0 | 0 | 1591 | 20 | 77 |
| Total PFHpA contamination per PWS in the county | 0 | 0 | 1484 | 28 | 104 |
| Total PFHxS contamination per PWS in the county | 0 | 0 | 1562 | 27 | 27 |
| Total of all PFAS contamination per PWS in the county | 0 | 0 | 1465 | 29 | 122 |
