# Supplementary material for: Peril in the Pipeline: Unraveling the threads of PFAS contamination in U.S. drinking water systems
Source: PLoS One. 2024 Apr 4;19(4):e0299789. doi: 10.1371/journal.pone.0299789 (PMC10994316; doi:10.1371/journal.pone.0299789)
Supplement: S4 Table — (DOCX) [file pone.0299789.s004.docx]

S4 Table. PFOA contamination hotspots.

| Hot Spots | state | County |
| --- | --- | --- |
| 1 | Alabama [18] | Etowah (9.53), DeKalb (9.02), Jackson (8.90), Marshall (8.81), Cherokee (8.02), Calhoun (7.89), Blount (7.03), Madison (7.00), St. Clair (6.89), Cullman (6.76), Morgan (5.78), Winston (5.27), Lawrence (5.06), Limestone (4.94), Walker (4.49), Talladega (4.36), Jefferson (3.83), Shelby (2.86) |
|  | Georgia  [33] | Chattooga (7.62), Floyd (7.38), Catoosa (7.38), Haralson (7.38), Walker (7.30), Whitfield (7.28), Dade (7.21), Gordon (7.19), Polk (7.19), Bartow (7.19), Paulding (6.93), Murray (6.85), Carroll (6.21), Cobb (5.80), Douglas (5.65), Cherokee (5.65), Gilmer (5.03), Coweta (4.66), Gwinnett (4.36), DeKalb (4.29), Hall (4.23), Forsyth (4.17), Fulton (4.17), Union (4.00), Dawson (4.00), Rockdale (3.15), Barrow (3.09), Fayette (3.04), Habersham (2.86), Stephens (2.61), Jackson (2.35), Henry (2.30), Walton (2.30) |
|  | Tennessee  [16] | Franklin (9.36), Marion (7.75), Hamilton (5.80), Meigs (5.70), McMinn (5.61), Lincoln (5.46), Bradley (5.46), Bledsoe (4.72), Rhea (4.65), Coffee (2.87), Lawrence (2.84), Giles (2.72), Monroe (2.65), Warren (2.40), Marshall (2.36), Maury (2.17) |
| 2 | New Jersey (18] | Ocean (7.42), Burlington (6.88), Mercer (6.72), Monmouth (6.72), Middlesex (6.72), Atlantic (6.43), Somerset (6.43), Hunterdon (6.08), Union (5.72), Hudson (5.71), Passaic (5.66), Bergen (5.66), Essex (5.62), Camden (5.39), Morris (5.37), Gloucester (5.21), Warren (4.92), Cape May (2.25), Marion (1.97) |
|  | Pennsylvania [13] | Philadelphia (6.50), Bucks (6.29), Montgomery (6.01), Lehigh (5.90), Northampton (5.89), Lackawanna (5.44), Pike (5.28), Carbon (5.19), Monroe (5.12), Delaware (5.03), Wayne (4.67), Chester (4.39), Berks (4.16) |
|  | New York [10] | Suffolk (6.26), Nassau (6.02), Queens (6.00), Rockland (5.48), Orange (5.10), Westchester (5.09), Putnam (5.00), Dutchess (4.95), Ulster (4.86), Sullivan (4.72) |
|  | Connecticut [2] | New Haven (3.00), Fairfield (2.97) |
| 3 | West Virginia [9] | Kanawha (2.47), Mason (2.33), Putnam (2.33), Cabell (2.16), Wood (2.10), Wayne (2.10), Lewis (2.05), Taylor (2.05) |
|  | Ohio [3] | Meigs (2.16), Washington (2.10), Athens (2.00) |
| 4 | Colorado [10] | Pueblo (8.76), Fremont (6.79), El Paso (5.99), Arapahoe (5.39),  Adams (4.93), Douglas (4.93), Denver (4.93), Broomfield (4.93), Jefferson (4.72), Gilpin (4.72), |
| 5 | Arizona [3] | Gila (3.73), Pinal (3.13), Graham (2.77) |

*Value in [ ] indicates number of counties that fall in the hot spot in respective states.*

*Value in ( ) indicates the z-score of Standardized Getis Ord statistics.*
