# Supplementary material for: Peril in the Pipeline: Unraveling the threads of PFAS contamination in U.S. drinking water systems
Source: PLoS One. 2024 Apr 4;19(4):e0299789. doi: 10.1371/journal.pone.0299789 (PMC10994316; doi:10.1371/journal.pone.0299789)
Supplement: S7 Table — (DOCX) [file pone.0299789.s007.docx]

S7 Table. PFHxS contamination hotspots

| Hot Spot | state | county |
| --- | --- | --- |
| 1 | New Jersey [18] | Atlantic (3.26), Monmouth (3.22), Middlesex (3.22), Mercer (2.72), Ocean (2.69), Union (2.68), Somerset (2.58), Cape May (2.50), Camden (2.47), Burlington (2.42), Hunterdon (2.41), Gloucester (2.37), Passaic (2.35), Bergen (2.35), Hudson (2.30), Essex (2.25), Morris (2.13), Cumberland (2.03) |
|  | Pennsylvania [8] | Bucks (2.69), York (2.61), Delaware (2.48), Chester (2.32), Philadelphia (2.28), Montgomery (2.28), Northampton (2.17), Lehigh (2.17) |
|  | Maryland [1] | Baltimore (2.01) |
|  | New York [1] | Queens (2.49) |
| 2 | Colorado [10] | Pueblo (13.96), Fremont (10.94), El Paso (9.73), Arapahoe (8.83), Adams (8.12), Douglas (8.12), Denver (8.12), Broomfield (8.12), Jefferson (7.82), Gilpin (7.82) |
| 3 | Wisconsin [7] | Oneida (3.94), Wood (3.21), Portage (2.97), Marinette (2.87), Marathon (2.87), Shawano (2.49), Waupaca (2.04) |
| 4 | Minnesota [3] | Beltrami (3.04), Itasca (2.26), Polk (2.26) |
| 5 | South Dakota [2] | Mellette (3.04), Hughes (3.04) |
| 6 | Kansas [2] | Ellis (2.02), Ford (2.02) |
| 7 | Texas [2] | Tom Green (2.02), Jones (2.02) |

*Value in [ ] indicates number of counties that fall in the hot spot in respective states.*

*Value in ( ) indicates the z-score of Standardized Getis Ord statistics.*
