# Supplementary material for: Peril in the Pipeline: Unraveling the threads of PFAS contamination in U.S. drinking water systems
Source: PLoS One. 2024 Apr 4;19(4):e0299789. doi: 10.1371/journal.pone.0299789 (PMC10994316; doi:10.1371/journal.pone.0299789)
Supplement: S9 Table — (DOCX) [file pone.0299789.s009.docx]

S9 Table. Tobit results for individual PFAS.

| VARIABLES - PFOA | Contaminants (Marginal effect) | | | | | |
| --- | --- | --- | --- | --- | --- | --- |
|  | PFOA | | PFOS | | PFHpA | |
|  | Model 1 | Model 2 | Model 1 | Model 2 | Model 1 | Model 2 |
| Size of the PWS – Small (1=small, 0= large) | -0.0000093***  (0.0000024) | -9.19E-06***  (2.39E-06) | -0.000009**  (0.00000376) | -0.000009**  (3.76E-06) | -0.00000060**  (0.00000028) | -6.00E-07**  (2.76E-07) |
| Source of water to the PWS- Surface (SW) | -0.0000092***  (0.0000028) | -9.66E-06***  (2.85E-06) | -0.000018***  (0.00000629) | -0.000018***  (6.42E-06) | 0.00000017  (0.00000017) | 9.92E-08  (1.58E-07) |
| Source of water to the PWS- Mixed (MX) | 0.0000013  (0.0000044) | 1.10E-06  (4.43E-06) | -0.000002  (0.00000669) | -0.000002  (6.70E-06) | 0.00000015  (0.00000049) | 9.83E-08  (4.91E-07) |
| Source of water to the PWS- Surface water-influenced ground water (SIG) | -0.0000022  (0.0000057) | -2.61E-06  (5.79E-06) | 0.000016*  (0.00000951) | 0.000016*  (9.53E-06) | -0.00000093  (0.00000090) | -9.76E-07  (9.25E-07) |
| Population log | 0.0000016  (0.0000013) | 1.64E-06  (1.29E-06) | 0.000012***  (0.00000424) | 0.000012***  (4.25E-06) | 0.00000074**  (0.00000034) | 7.62E-07**  (3.46E-07) |
| Non-White population (%) | -0.0000004***  (0.0000001) | -3.95E-07***  (1.30E-07) | -0.000001***  (3.32E-07) | -0.000001***  (3.42E-07) | -0.00000004**  (0.00000002) | -4.6508**  (2.17E-08) |
| Poverty (%) | 0.0000004  (0.0000004) |  | -4.44E-07  (6.43E-07) |  | 0.00000001  (0.00000003) |  |
| Nonwhite Poverty (%) | -0.0000003  (0.0000002) |  | -2.85E-08  (3.24E-07) |  | -0.00000002  (0.00000002) |  |
| Regional Price Parities (RPP) adjusted per capita income (USD) |  | -2.94E-10  (1.94E-10) |  | 9.69E-11  (3.29E-10) |  | -2.92E-11  (2.19E-11) |
| Log Housing density (house/sq mil) | 0.0000059***  (0.0000019) | 6.51E-06***  (2.01E-06) | 0.000006**  (0.00000282) | 0.000006**  (2.95E-06) | -0.00000009  (0.00000013) | -1.67E-08  (1.33E-07) |
| Percentage Contribution to the GDP from |  |  |  |  |  |  |
| Agriculture | -0.0000007**  (0.0000004) | -8.85E-07  (4.16E-07) | -1.63E-07  (5.08E-07) | -3.50E-07  (4.93E-07) | -0.00000001  (0.00000003) | -2.82E-08  (3.25E-08) |
| Durable goods manufacturing | -0.0000001  (0.0000002) | -2.26E-07  (1.99E-07) | -2.11E-07  (3.51E-07) | -2.24E-07  (3.54E-07) | -0.00000008**  (0.00000004) | -8.59E-08**  (4.12E-08) |
| Non-durable good manufacture | 0.0000008***  (0.0000002) | 7.99E-07***  (2.20E-07) | 0.000001***  (4.78E-07) | 0.000001***  (4.71E-07) | 0.00000005*  (0.00000002) | 4.06E-08*  (2.24E-08) |
| Healthcare and social assistance | 0.0000006*  (0.0000003) | 3.33E-07  (2.80E-07) | 0.000002***  (7.43E-07) | 0.000002***  (7.09E-07) | 0.00000006**  (0.00000004) | 4.47E-08  (3.01E-08) |
| Food and accommodation | 0.0000002  (0.0000004) | 5.93E-08  (3.67E-07) | 0.000003***  (0.00000111) | 0.000003***  (1.09E-06) | 0.00000004  (0.00000004) | 3.38E-08  (4.28E-08) |
| Government enterprise | 0.0000004***  (0.0000002) | 3.61E-07***  (1.48E-07) | 0.000001***  (3.53E-07) | 0.000001***  (3.49E-07) | 0.00000006**  (0.00000003) | 4.86E-08**  (2.29E-08) |
| Observations | 35,589 | 35,589 | 35,589 | 35,589 | 35,589 | 35,589 |
| Pseudo R2 | 0.3441 | 0.3441 | 0.2184 | 0.2179 | 0.4504 | 0.4501 |
| Prob > chi2 | 0 | 0.000 | 0 | 0.00000 | 0 |  |
| AIC | 1740.996 | 1739.025 | 2187.249 | 2186.55 | 885.7002 | 884.0773 |
| BIC | 2122.586 | 2112.136 | 2543.401 | 2534.22 | 1224.892 | 1214.789 |

*Standard errors in parentheses*

**** p<0.01, ** p<0.05, * p<0.1*

Tobit outputs are for the marginal effect of the censored sample

*Model 1 is model with poverty as independent variable*

*Model 2 is model with income as independent variable*
