# Supplementary material for: Peril in the Pipeline: Unraveling the threads of PFAS contamination in U.S. drinking water systems
Source: PLoS One. 2024 Apr 4;19(4):e0299789. doi: 10.1371/journal.pone.0299789 (PMC10994316; doi:10.1371/journal.pone.0299789)
Supplement: S10 Table — (DOCX) [file pone.0299789.s010.docx]

S10 Table. Probit results for individual PFAS.

|  | Contaminants (Marginal Effect) | | | | | |
| --- | --- | --- | --- | --- | --- | --- |
|  | PFOA | | PFOS | | PFHpA | |
|  | Model 1 | Model 2 | Model 1 | Model 2 | Model 1 | Model 2 |
| Size of the PWS – Small (1=small, 0= large) | -0.0045854***  (0.0006519) | -0.00454***  (0.0007) | -0.00244***  (0.0007582) | -0.0024***  (0.000757) | -0.0025***  (0.0004785) | 0.002***  (0.0005) |
| Source of water to the PWS- Surface (SW) | -0.0037098***  (0.0007919) | -0.00387***  (0.0008) | -0.00404***  (0.0007939) | -0.0042***  (0.0007962) | 0.00065  (0.0005254) | 0.0004  (0.0005) |
| Source of water to the PWS- Mixed (MX) | 0.0007486  (0.001814) | 0.00066  (0.0018) | -0.00002  (0.00151) | -3.13E-05  (0.0015093) | 0.00079  (0.001628) | 0.0006  (0.0016) |
| Surface water-influenced ground water (SIG) | -0.0003867  (0.0023285) | -0.00062  (0.0023)) | 0.00419**  (0.0018761) | 0.0042**  (0.0018776) | -0.00312  (0.0027917) | -0.0032  (0.0027) |
| Population log | 0.0006792  (0.0005107) | 0.00063  (0.0005) | 0.00293***  (0.0005201) | 0.0029***  (0.0005243) | 0.00251***  (0.0004814) | 0.0025***  (0.0005) |
| Non-White population (%) | -0.0001513***  (0.0000425) | -0.00016***  (0.0000) | -0.00022***  (0.0000455) | -0.0002***  (0.0000427) | -0.00015***  (0.0000336) | -0.0002***  (0.00003) |
| Poverty (%) | 0.0000996  (0.0001565) |  | -0.00011  (0.0001452) |  | 0.000001  (0.0001182) |  |
| Nonwhite Poverty (%) | -0.000073  (0.0000832) |  | 0.00000  (0.0000751) |  | -0.00006  (0.0000648) |  |
| Regional Price Parities (RPP) adjuster per capita income (USD) |  | 0.00000**  (0.0000) |  | -7.30E-09  (7.60E-08) |  | -1.05E-07*  (6.09e-08) |
| Log Housing density (house/sq mil) | 0.0025839***  (0.0005679) | 0.00286***  (0.0006) | 0.00154***  (0.0004776) | 0.0016***  (0.0005054) | -0.00023  (0.0004366) | 2.48E-05  (0.0004) |
| Percentage Contribution to the GDP from |  |  |  |  |  |  |
| Agriculture | -0.0002824**  (0.0001537) | -0.00034**  (0.0002) | -0.00003  (0.0001161) | -0.0001  (0.0001106) | -0.00003  (0.0001035) | -0.0001  (0.0001) |
| Durable goods manufacturing | -0.00005  (0.0000784) | -0.00008  (0.0001) | -0.00004  (0.0000792) | -4.13E-05  (0.0000794) | -0.00026***  (0.0000698) | -0.0003***  (0.0001) |
| Non-durable good manufacture | 0.0003479***  (0.0000597) | 0.00032***  (0.0001) | 0.00034***  (0.0000575) | 0.0003***  (0.0000582) | 0.00017***  (0.0000491) | 0.0001***  (0.00005) |
| Healthcare and social assistance | 0.0002333**  (0.0001113) | 0.00014  (0.0001) | 0.00050***  (0.0000997) | 0.0005***  (0.0000992) | 0.00023***  (0.0000786) | 0.0002**  (0.0001) |
| Food and accommodation | 0.0000501  (0.0001514) | 0.00002  (0.0002) | 0.00077***  (0.0001371) | 0.0008***  (0.0001363) | 0.00014  (0.0001358) | 0.0001  (0.0001) |
| Government enterprise | 0.0001847***  (0.0000508) | 0.00015***  (0.0001) | 0.00024****  (0.0000484) | 0.0002***  (0.0000503) | 0.00020***  (0.0000369) | 0.0002***  (0.00004) |
| Observations | 28,908 | 28,908 | 27,667 | 27,667 | 25,844 | 25,844 |
| Pseudo R2 | 0.1755 | 0.1764 | 0.1469 | 0.1466 | 0.1947 | 0.1950 |
| Prob > chi2 | 0 | 0.00000 | 0 | 0.00000 | 0 |  |
| AIC | 3403.389 | 3397.97 | 2712.366 | 2711.52 | 2180.663 | 2177.895 |
| BIC | 3767.352 | 3753.66 | 3049.714 | 3040.64 | 2498.897 | 2487.968 |

*Standard errors in parentheses*

**** p<0.01, ** p<0.05, * p<0.1*
