# Supplementary material for: Peril in the Pipeline: Unraveling the threads of PFAS contamination in U.S. drinking water systems
Source: PLoS One. 2024 Apr 4;19(4):e0299789. doi: 10.1371/journal.pone.0299789 (PMC10994316; doi:10.1371/journal.pone.0299789)
Supplement: S1 Fig — Panel A, B, C, and D show the number of PFOA, PFOS, PFHpA, and PFHxS contaminated samples, respectively. (DOCX) [file pone.0299789.s011.docx]

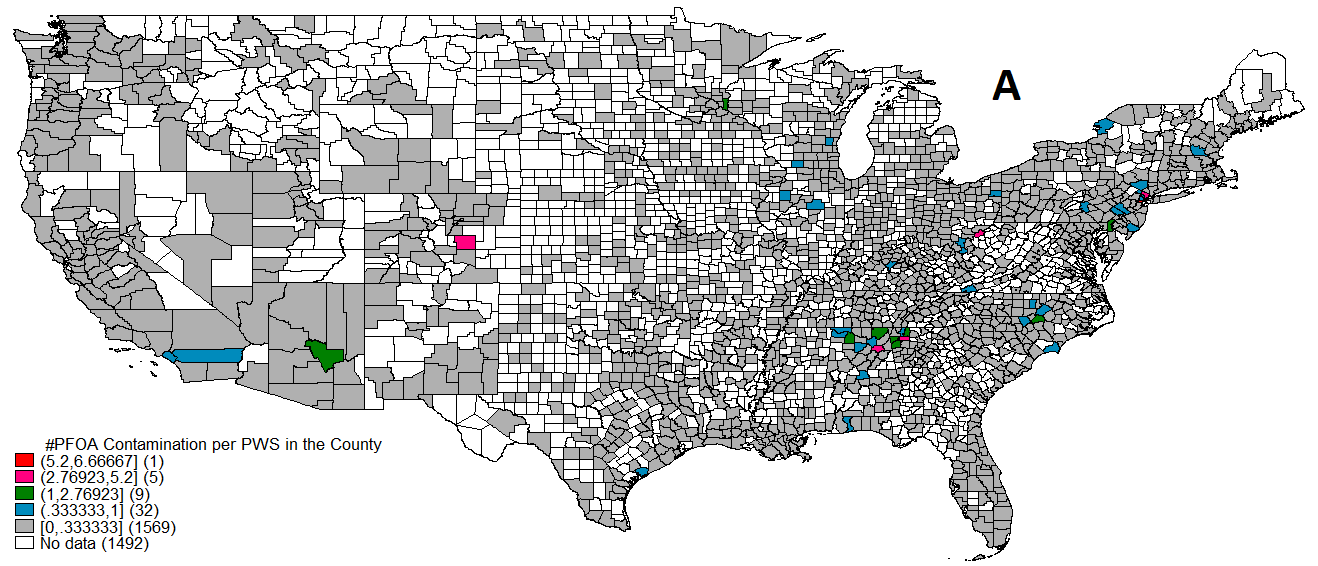


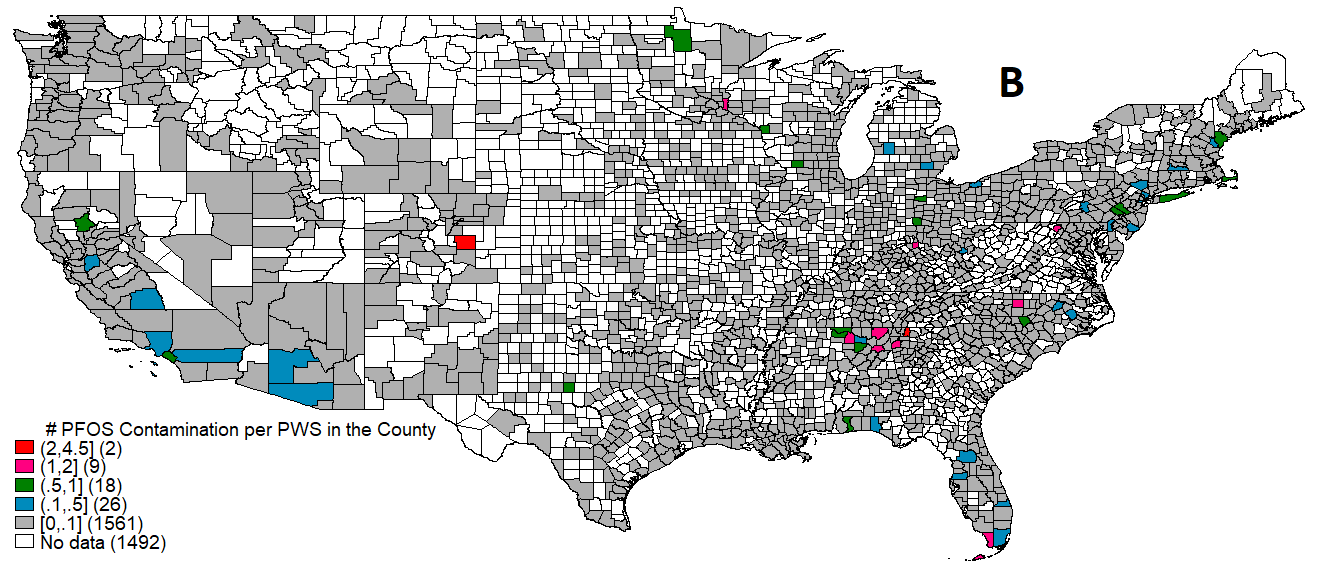


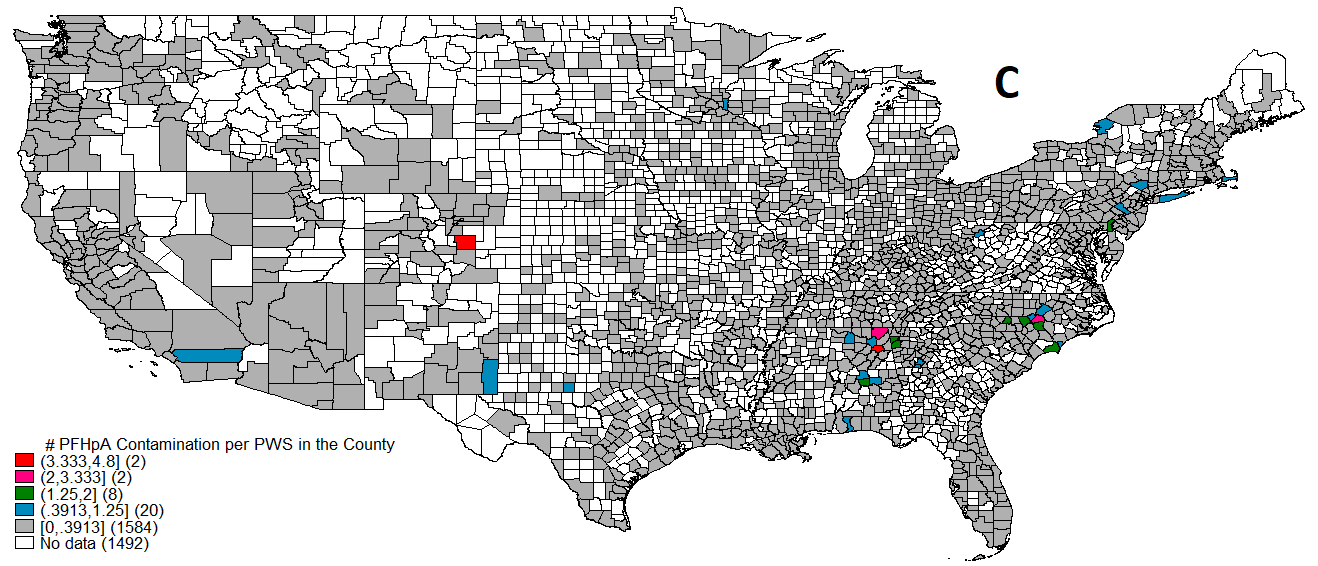


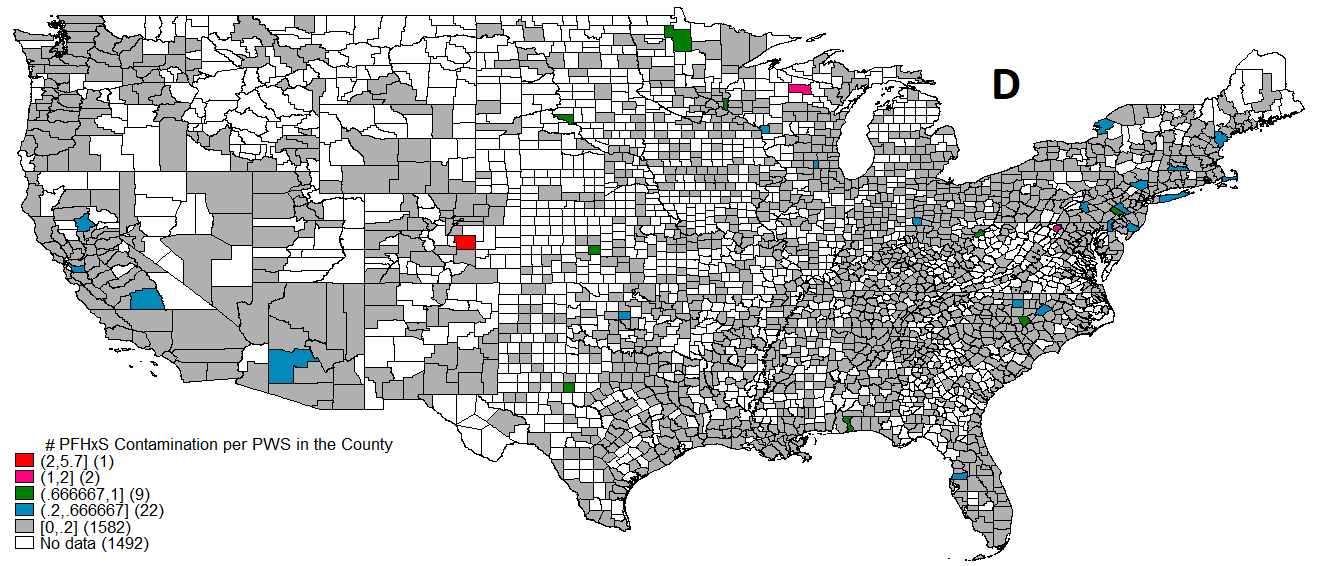


S1 Fig. Number of different types of PFAS contaminated water samples per PWS and county.

Panels A, B, C, and D show the number of PFOA, PFOS, PFHpA, and PFHxS contaminated samples, respectively.
