# Supplementary material for: Peril in the Pipeline: Unraveling the threads of PFAS contamination in U.S. drinking water systems
Source: PLoS One. 2024 Apr 4;19(4):e0299789. doi: 10.1371/journal.pone.0299789 (PMC10994316; doi:10.1371/journal.pone.0299789)
Supplement: S2 Fig — Panel (A) (B) (C), and (D) shows the hotspots of PFOA, PFOS, PFHpA and PFHxS, respectively. Intervals in the legends are selected based on 1% and 5% levels of significance. (DOCX) [file pone.0299789.s012.docx]

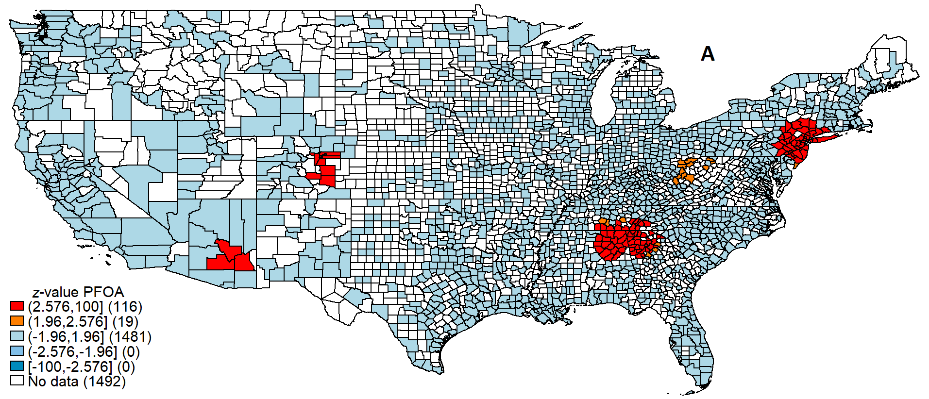


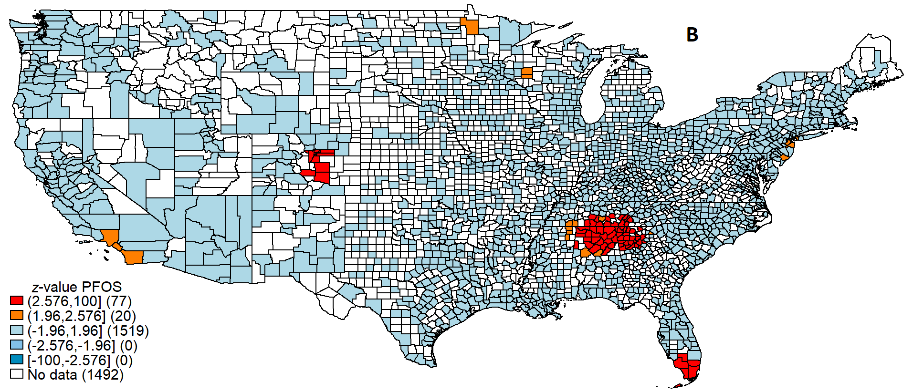


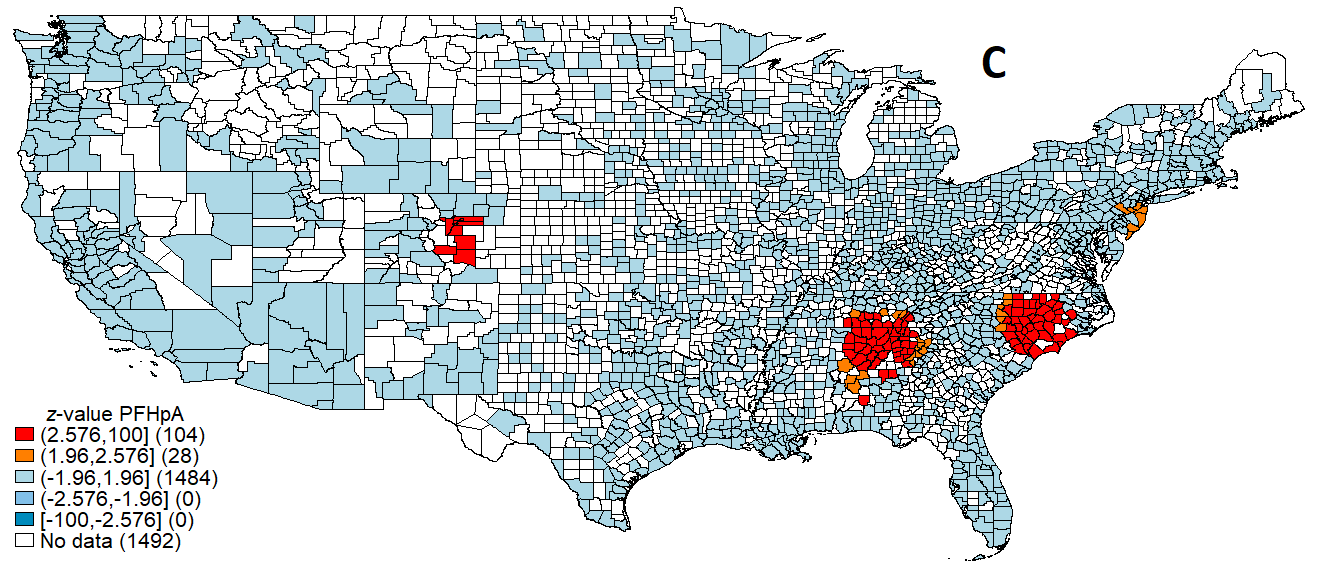


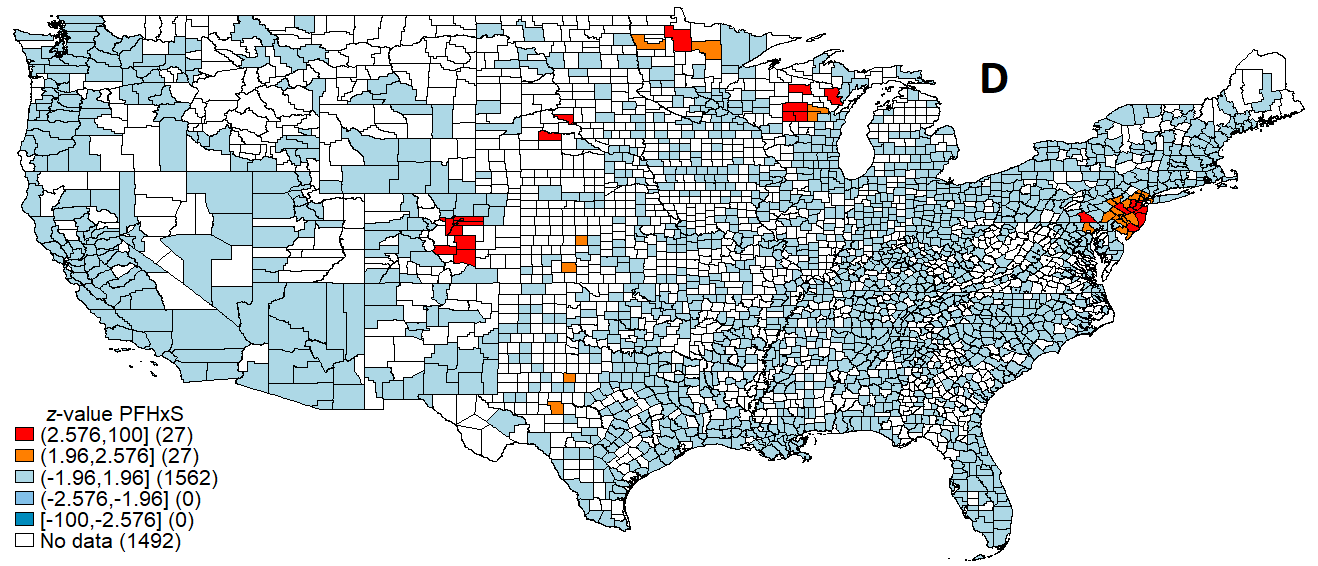


S2 Fig. Hot spot of different types of PFAS contaminations.

Panels A, B, C, and D show the hotspots of PFOA, PFOS, PFHpA and PFHxS, respectively. Intervals in the legends are selected based on 1% and 5% levels of significance.
